# RUNX1 promotes tumour metastasis by activating the Wnt/β-catenin signalling pathway and EMT in colorectal cancer

**DOI:** 10.1186/s13046-019-1330-9

**Published:** 2019-08-01

**Authors:** Qingyuan Li, Qiuhua Lai, Chengcheng He, Yuxin Fang, Qun Yan, Yue Zhang, Xinke Wang, Chuncai Gu, Yiqing Wang, Liangying Ye, Lu Han, Xin Lin, Junsheng Chen, Jianqun Cai, Aimin Li, Side Liu

**Affiliations:** 1 Guangdong Provincial Key Laboratory of Gastroenterology, Department of Gastroenterology, Nanfang Hospital, Southern Medical University, No. 1838, Guangzhou Avenue North, Guangzhou, Guangdong People’s Republic of China; 2 Department of Pathology, Nanfang Hospital, Southern Medical University, Guangzhou, China; 3 Department of Pathology, School of Basic Medical Sciences, Southern Medical University, Guangzhou, China

**Keywords:** RUNX1, Colorectal cancer, KIT, Wnt/β-catenin, EMT

## Abstract

**Background:**

Runt-related transcription factor 1 (RUNX1) plays the roles of an oncogene and an anti-oncogene in epithelial tumours, and abnormally elevated RUNX1 has been suggested to contribute to the carcinogenesis of colorectal cancer (CRC). However, the mechanism remains unclear.

**Methods:**

The expression of RUNX1 in CRC and normal tissues was detected by real-time quantitative PCR and Western blotting. The effect of RUNX1 on CRC migration and invasion was conducted by functional experiments in vitro and in vivo. Chromatin Immunoprecipitation assay verified the direct regulation of RUNX1 on the promoter of the KIT, which leads to the activation of Wnt/β-catenin signaling.

**Results:**

RUNX1 expression is upregulated in CRC tissues. Upregulated RUNX1 promotes cell metastasis and epithelial to mesenchymal transition (EMT) of CRC both in vitro and in vivo. Furthermore, RUNX1 can activate Wnt/β-catenin signalling in CRC cells by directly interacting with β-catenin and targeting the promoter and enhancer regions of KIT to promote KIT transcription. These observations demonstrate that RUNX1 upregulation is a common event in CRC specimens and is closely correlated with cancer metastasis and that RUNX1 promotes EMT of CRC cells by activating Wnt/β-catenin signalling. Moreover, RUNX1 is regulated by Wnt/β-catenin.

**Conclusion:**

Our findings first demonstrate that RUNX1 promotes CRC metastasis by activating the Wnt/β-catenin signalling pathway and EMT.

**Electronic supplementary material:**

The online version of this article (10.1186/s13046-019-1330-9) contains supplementary material, which is available to authorized users.

## Background

Colorectal cancer (CRC) is one of the most common human malignancies and is a leading cause of cancer-related death worldwide [1]. Wnt/β-catenin signalling plays a vital role during development and in maintaining homeostasis in multiple tissues throughout the body, but Wnt/β-catenin signalling also plays an essential role in the initiation and early progression, as well as in later stages of invasion and metastasis, of CRC [2–5]. The process known as epithelial to mesenchymal transition (EMT) is a key mechanism in the invasiveness and metastatic drive in most cases of carcinoma [6]. Moreover, dysregulation of the Wnt/β-catenin signalling pathway has been shown to play a significant role in the EMT required for colorectal tumour metastasis [7, 8].

Runt-related transcription factor 1 (RUNX1), also called acute myeloid leukaemia 1, is a member of RUNX family of transcription factors (RUNX1, RUNX2 and RUNX3), and this family is composed of evolutionarily conserved transcription factors that function as critical lineage determinants in various tissues [9–11]. RUNX1 is one of the most frequently mutated genes in a variety of haematological malignancies and has been proposed to play tumour suppressor roles in leukaemia; however, more recent studies suggest that wild-type RUNX1 is required for the growth and survival of certain types of leukaemia cells [12, 13]. Additionally, RUNX1 plays the role of an oncogene and an anti-oncogene in epithelial tumours, and its oncogenic effect has been given increasing attention [10, 14–19].

In previous studies, RUNX1 and the Wnt/β-catenin signalling pathway were shown to be closely related, and we found that their relationship involved inhibition [18] or interdependence [17, 20–23]. The role of RUNX1 in CRC has been examined in several studies [24, 25], and its possible mechanism involves inducing EMT by regulating the Wnt signalling pathway.

In the present study, we determined that the expression of RUNX1 was significantly upregulated in CRC tissues compared to its expression in adjacent tissues. We showed that RUNX1 is a critical activator of CRC cell metastasis both in vitro and in vivo. Moreover, RUNX1 activated the Wnt/β-catenin signalling pathway to promote EMT and tumour metastasis.

## Materials and methods

### Clinical specimens

A total of 73 patients who underwent radical operation for CRC at Nanfang Hospital of Southern Medical University were included in the study after obtaining informed consent. A diagnosis of CRC was histopathologically confirmed for each patient sample. Cancer tissues and matched normal tissues were stored at -80C° for quantitative real-time PCR (qRT-PCR) and Western blotting analyses. Additionally, none of the patients had received radiotherapy or chemotherapy before surgery. The protocols used in this study were approved by Nanfang Hospital’s Protection of Human Subjects Committee.

### Cell culture, plasmid construction, lentiviral construction and cell transfection

Human embryonic kidney cell line (293 T), human normal colon epithelial cell line (FHC) and six human CRC cell lines (SW480, SW620, RKO, HCT116 HT29 and LOVO) were purchased from the Cell Bank of Type Culture Collection (CBTCC, Chinese Academy of Sciences, Shanghai, China) and were cultured in Dulbecco’s modified Eagle medium (DMEM) (Gibco, Carlsbad, CA) supplemented with 10% fetal bovine serum (FBS; Gibco, Carlsbad, CA). Cells were maintained at 37 °C in a humidified 5% CO_2_ atmosphere.

### Plasmid construction, lentiviral construction and cell transfections

RUNX1 overexpression and knockdown were performed using a lentiviral packaging system. To construct overexpressing exogenous and RNA-interfered endogenous RUNX1 cell lines, full-length RUNX1 (NM_001754) was cloned into the expression vector pLenti-EF1a-EGFP-P2A-Puro-CMV (Obio Technology, Shanghai, China) and transfected into HCT116 and RKO cell lines according to the manufacturer’s instructions. Knockdown of endogenous RUNX1 was mediated by designed shRNAs (Cyagen, Guangzhou, China) that were transfected into SW480 and RKO cell lines according to the manufacturer’s instructions. The RUNX1 shRNA sequence was sense 5′-CCAGGTTGCAAGATTTAAT-3′ and 5′-GGCAGAAACTAGATGATCA-3′, and the scramble sequence was sense 5′-CCTAAGGTTAAGTCGCCCTCG-3′. Transduced cells were selected in medium containing puromycin (#EZ2811D376, BioFroxx, Germany) (2 μg/ml) and maintained in medium containing puromycin (1 μg/ml).

### RNA isolation and qRT-PCR

Total RNA was extracted from cells or tissues with TRIzol reagent (TaKaRa, China). qRT-PCR was performed using the PrimeScript RT Reagent Kit (#RR035A, TaKaRa, China) and SYBR Premix Ex Taq (#RR820A, TaKaRa, Dalian, China) following the manufacturer’s instructions. Our results were normalized to the expression of glyceraldehyde-3-phosphate dehydrogenase (GAPDH) or U6. The specific primers used are listed in Additional file 5: Table S1. The qRT-PCR results were analyzed to obtain the Ct values of the amplified products, and data were analyzed by the 2-ΔΔCt method.

### Western blot and immunohistochemistry (IHC) analysis

We performed Western blotting according to the methods of a previous study. Protein lysates were prepared, subjected to sodium dodecyl sulfate polyacrylamide gel electrophoresis (SDS-PAGE), transferred onto polyvinylidene difluoride (PVDF) membranes and blotted according to standard methods using the following antibodies: RUNX1 (#4336, CST), KIT (#3074, CST), CD44 (#3570, CST), cyclin D1 (#2978, CST), c-Jun (#9165, CST), LEF1 (#2230, CST), Met (#8198, CST), c-Myc (#5605, CST), TCF1/TCF7 (#2230, CST), β-catenin (#8480, CST), E-cadherin (#3195, CST), N-cadherin (#13116, CST), Vimentin (#5741, CST), TCF8/ZEB1 (#3396, CST), AXIN1 (#2087, CST) and GAPDH (60004–1-Ig, Proteintech).

Immunohistochemistry was performed following the manufacturer’s instructions (PV-6001, ZSGB-BIO, Beijing, China) using the E-cadherin antibody (60335–1-Ig, Proteintech), N-cadherin (66219–1-Ig, Proteintech), and Vimentin (60330–1-Ig, Proteintech). One independent pathologist used software ImageJ to calculate gray values for pathological scoring.

### Cell Transwell migration and invasion assays

Approximately 2 × 10^5^ cells were suspended in 100 μl serum-free medium and seeded in the upper 8-μm-pore Transwell chambers (3422, Corning, USA), and the lower chambers were filled with 500 μL of 20% FBS medium. For the cell invasion assay, the Transwell chambers were Matrigel-coated (354234, Corning, USA). Then, the cells were incubated at 37 °C for 24–48 h to allow for migration or invasion. For quantification, the cells were fixed with 4% paraformaldehyde for 20 min, stained with hematoxylin for 20 min at room temperature, and counted in five randomly chosen fields (× 200) under a microscope.

### Cell wound healing assay

Cells were seeded on six-well culture plates at a density of approximately 1 × 10^6^ cells per well and incubated for 24 h (80–90% confluence). Scratch wounds were produced by a 10 μl plastic pipette tip, and then cells were cultured in DMEM with 2% FBS. Wound margins were photographed, and migration was monitored after 48 h of wound formation. Cell motility was quantified by measuring the distance between the advancing margins of cells in three randomly selected microscopic fields (× 200) at each time point.

### Immunofluorescence

For immunofluorescence, cells were seeded on cover slips. Overexpression or shRNA knockdown treatment was performed after 24 h. After the indicated treatment, the cells were cultured for 48 h, fixed with 4% formaldehyde for 10 min at room temperature, and washed three times with wash buffer (0.02% Tween 20/PBS). Then, the cells were permeabilized with 0.5% Triton X-100/PBS for 10 min at room temperature. The cells were washed three times with wash buffer (5 min each time) and then incubated with 1.5% bovine serum albumin (BSA)/phosphate-buffered saline (PBS) solution (blocking solution) for 30 min at room temperature. The E-cadherin antibody (60335–1-Ig, Proteintech), N-cadherin (66219–1-Ig, Proteintech), Vimentin (60330–1-Ig, Proteintech), and β-catenin (66379–1-Ig, Proteintech) were incubated in blocking solution at 4 °C overnight. Rhodamine phalloidin (1:1500, Cytoskeleton, PHDR1), which was used for detecting F-actin, was incubated in blocking solution at room temperature in the dark for 60 min. After washing, the cells were incubated with Alexa594-conjugated secondary antibodies for 60 min at room temperature in a protected environment in the dark (Life Technologies, A-21235, 1:500 in blocking buffer) followed by counterstaining with DAPI (Thermo Fisher). Samples were mounted with ProLong Gold antifade reagent (Life Technologies) and imaged on a confocal microscope (× 200).

### Chromatin immunoprecipitation

ChIP assays were performed using a kit (#17–10085, Merck, German), and all the experimental procedures were performed according to the manufacturer’s instructions. Briefly, cells (1 × 10^7^) in a 15-cm culture dish were treated with 1% formaldehyde to cross-link chromatin-associated proteins to DNA. The cell lysates were subjected to ultrasound for 9–10 sets of 10-s pulses at 40% output to shear the DNA into fragments between 200 and 1000 bps. Equal cell lysates were incubated with 10 μl of DYKDDDDK Tag (bound to the same epitope as the Sigma Anti-Flag M2 antibody) (#14793, CST), anti-IgG antibody (Merck) as a negative control and anti-RNA polymerase II as a positive control. All the above chromatin supernatants were incubated with 20 μL magnetic protein A/G beads overnight at 4 °C with rotation. On the second day, the protein-DNA complexes were reversed and purified for pure DNA. The human c-Kit binding sites were amplified with qRT-PCR and PCR. The specific primers used are listed in Additional file 6: Table S2.

### Orthotopic injection metastatic mouse model

Female athymic 4 to 5-week-old BALB/c nude mice were purchased from the Laboratory Animal Services Centre of Guangdong Province and were maintained in a specific pathogen-free facility. For the orthotopic injection metastatic mouse model assay, the nude mice of the experimental group were anesthetized with 1% pentobarbital, and 5 × 10^6^ cells were injected under the ileocecal serosa. The general growth conditions in the nude mice were observed after surgery to determine whether there were signs of cachexia, such as wasting and arched backs. Then, 40 days after the operation, all mice were sacrificed, and the intestines, liver, spleen and lungs were removed for photographing. After formalin fixation, paraffin sections were generated and stained with hematoxylin and eosin (H&E).

### Statistical analysis

The SPSS 22.0 (IBM; Chicago, IL, USA) and Microsoft Excel 2016 (Microsoft, Redmond, WA, USA) statistical analysis software programs were used for statistical analysis of the experimental data. The significance of differences between groups was estimated by Student’s t-test. Additionally, multiple group comparisons were analyzed with one-way ANOVA. Bivariate correlations between study variables were calculated by Spearman’s rank correlation coefficients. * *P* < 0.05, ** *P* < 0.01, and *** *P* < 0.001 were considered significant.

## Results

### RUNX1 is upregulated in CRC

**Fig. 1:**
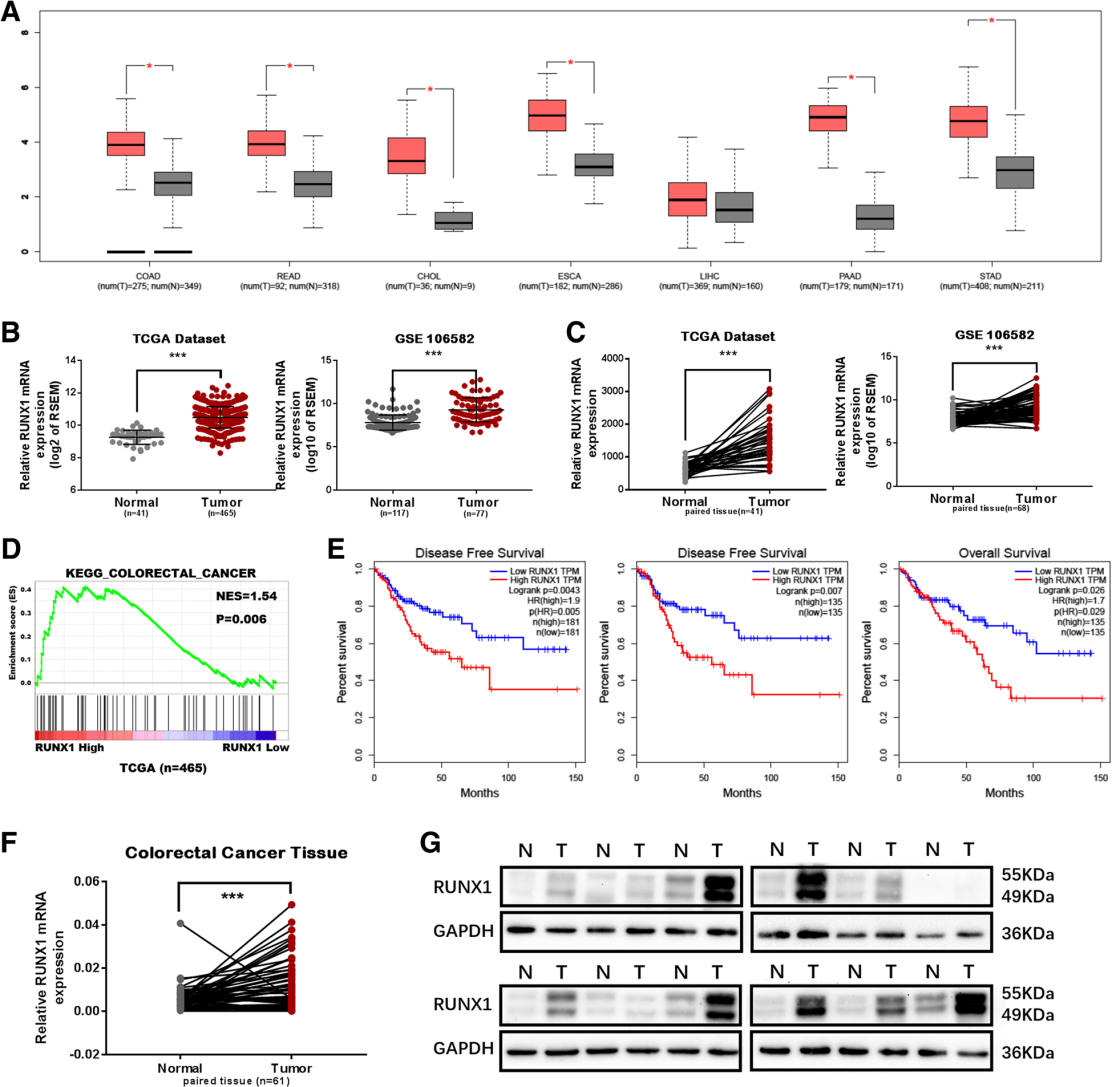
RUNX1 expression in colorectal cancer tissues. **a** RUNX1 is highly expressed in the majority of gastrointestinal tumors (TCGA database online website GEPIA: http://gepia.cancer-pku.cn/). **b** The expression level of RUNX1 mRNA in colorectal cancer is higher than that in normal tissues. (Database source: TCGA (*n* = 465) and GSE106582 (*n* = 117)). **c** The expression level of RUNX1 mRNA in colorectal cancer is higher than that in the paired normal tissues. (Database source: TCGA (*n* = 41) and GSE106582 (*n* = 68)). **d** Positive correlation between colorectal cancer and RUNX1 expression was shown using TCGA (n = 465). **e** Percent of disease free survival and overall survival with low RUNX1 expression was higher than that with high RUNX1 expression, given by GEPIA. **f** mRNA expression of RUNX1 detected by qRT-PCR in colorectal cancer is higher than that in the paired non-cancerous tissues (*n* = 61). **g** RUNX1 protein expression in 12 pairs of colorectal cancer tissues is higher that than in adjacent non-tumor tissues

An analysis of The Cancer Genome Atlas (TCGA) database using the online Gene Expression Profiling Interactive Analysis (GEPIA) tool (http://gepia.cancer-pku.cn/) indicated that RUNX1 is highly expressed in most types of digestive system tumours (Fig. 1a). To screen CRC gene expression profiles, we selected two qualified gene expression microarray datasets (TCGA and GSE106582) (Fig. 1b) and determined that RUNX1 was differentially expressed in colorectal tumour tissues compared to its expression in non-neoplastic tissues (*p* < 0.001) in two independent samples and in paired samples (Fig. 1c). An analysis of the correlation between CRC-related gene set enrichment and RUNX1 expression showed statistically significant (NES = 1.54, *p* = 0.006) results in TCGA database, and Gene Set Enrichment Analysis (GSEA) (http://software.broadinstitute.org/gsea/index.jsp) suggested a significant correlation between RUNX1 expression and these genes (Fig. 1d). Notably, Kaplan-Meier survival analysis revealed that patients with high RUNX1 expression levels had poorer disease-free survival and overall survival than patients with low RUNX1 expression levels. Similar results were found when the GEPIA web server was used to analyze TCGA database (Fig. 1e) as well as in the PROGgeneV2 - Pan Cancer Prognostics Database using the GEO database (http://genomics.jefferson.edu/proggene/) (Additional file 1: Figure S1A and B).

Furthermore, to determine whether RUNX1 protein expression is also increased in CRC, qRT-PCR and Western blotting were performed in 61 pairs of fresh samples and in 12 pairs of fresh samples, including cancers and matched adjacent normal tissues or independent normal tissues (Fig. 1f and g). In this analysis, the RUNX1 levels were significantly upregulated in most CRC tissues. In the patient clinical information statistics table (Additional file 7: Table S3), we found that patients with high expression of RUNX1 were worse in AJCC stage, T stage and M stage, when the patients were divided into high and low RUNX1 expression groups by the median expression of RUNX1 mRNA. Western blotting and qRT-PCR analysis revealed varying levels of RUNX1 expression in six CRC cell lines: SW480, SW620, HT29, HCT116, RKO and LoVo. RUNX1 expression was highest in SW480 and SW620 cells; lowest in HCT116, HT29 and LoVo cells; and moderately expressed in RKO cells (Additional file 1: Figure S1C).

### RUNX1 promotes CRC cell metastasis in vitro and in vivo

**Fig. 2:**
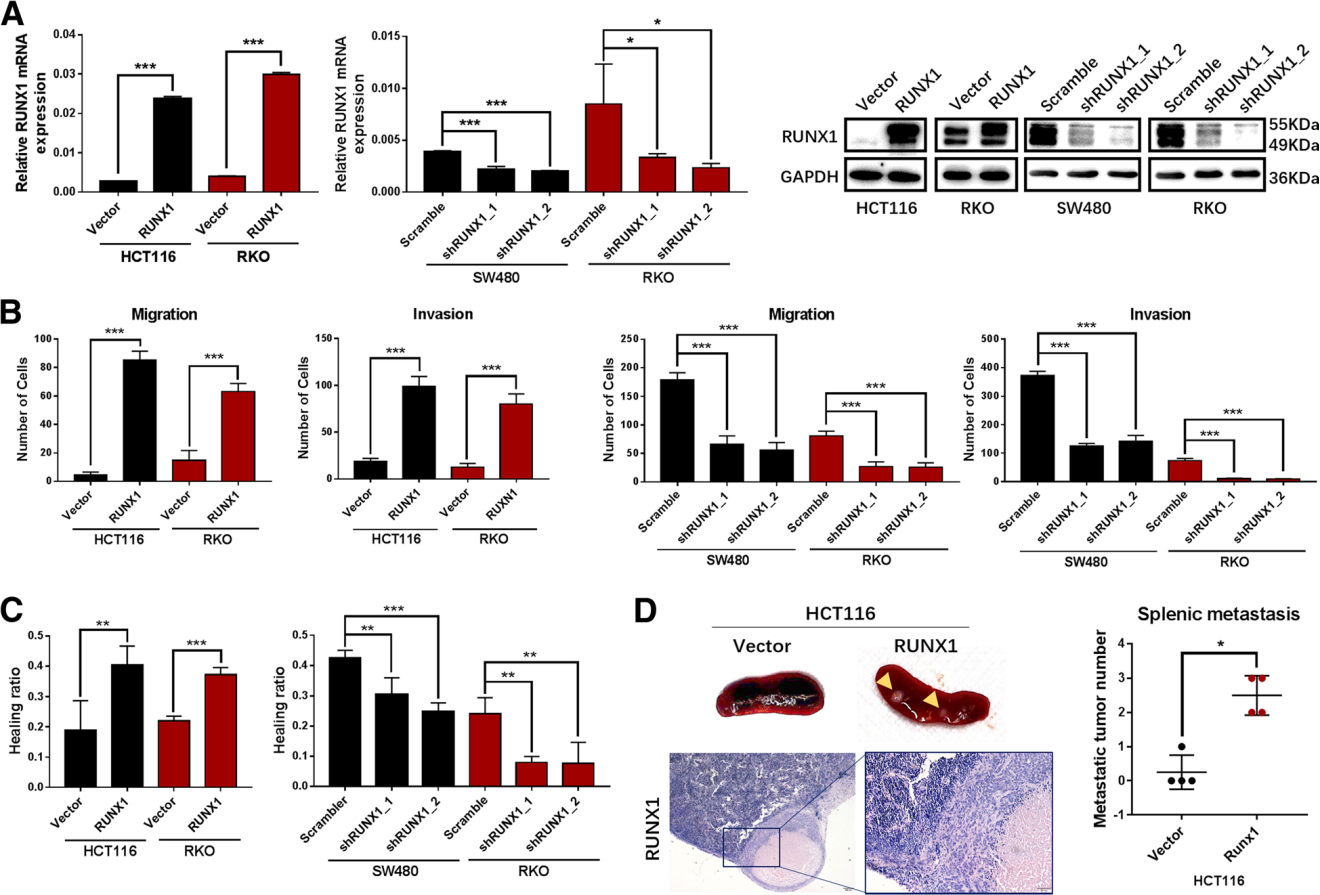
RUNX1 affects the progression and metastasis of colorectal cancer in vitro and vivo. **a** Stable RUNX1-expression and silencing cell lines were established in HCT116, RKO and SW480 cells. Expression of RUNX1 mRNA and protein detected by qPCR and western blot analysis in HCT116, RKO and SW480 cells with RUNX1 overexpressed and silencing. **b** Migration and invasion ability detected by Transwell assays in HCT116, RKO and SW480 cells with RUNX1 overexpressed and silencing. **c** Migration and invasion ability detected by Wound healing in HCT116, RKO and SW480 cells with RUNX1 overexpressed or silencing. **d** General form of splenic metastasis and HE staining in HCT116/Vector and HCT116/RUNX1 group. Metastatic tumor number of splenic metastasis was also compared

To investigate the biological function of RUNX1 in CRC cells in vitro and in vivo, we established stable RUNX1-overexpressing CRC cell lines with HCT116 and RKO cells and RUNX1-silenced cell lines using a lentiviral vector carrying two specific shRNAs in SW480 and RKO cells. Forty-eight hours after transfection, qRT-PCR and Western blotting analysis showed a significant increase in RUNX1 mRNA and protein expression in HCT116-RUNX1 and RKO-RUNX1 cells and a significant decrease in RUNX1 expression in SW480-shRUNX1_1, SW480-shRUNX1_2, RKO-shRUNX1_1 and RKO-shRUNX1_2 cells compared to the expression levels in control cells (Fig. 2a).

Markedly faster wound closure was observed in HCT116-RUNX1 and RKO-RUNX1 cells, but SW480-shRUNX1_1, SW480-shRUNX1_2, RKO-shRUNX1_1 and RKO-shRUNX1_2 cells exhibited slower wound closure, as revealed by a wound healing assay (Fig. 2c and Additional file 2: Figure S2B). Moreover, the results from Transwell assays revealed that overexpression of RUNX1 significantly increased the migration and invasion abilities of CRC cells. Next, our results showed that the migration and invasion abilities were significantly decreased in cells with RUNX1 downregulation (Fig. 2b and Additional file 2: Figure S2A).

Furthermore, we also achieved consistent results for in vivo metastasis assays. As shown by our results, tumours formed by cells overexpressing RUNX1 showed stronger metastatic ability, and more tumours were formed in the spleen (Additional file 2: Figure S2C) in the nude mice cecum tumour orthotopic model transfected with RUNX1-overexpressing CRC HCT116-RUNX1 cells than in the control group transfected with the HCT116-vector (Fig. 2d).

### RUNX1 promotes EMT

**Fig. 3:**
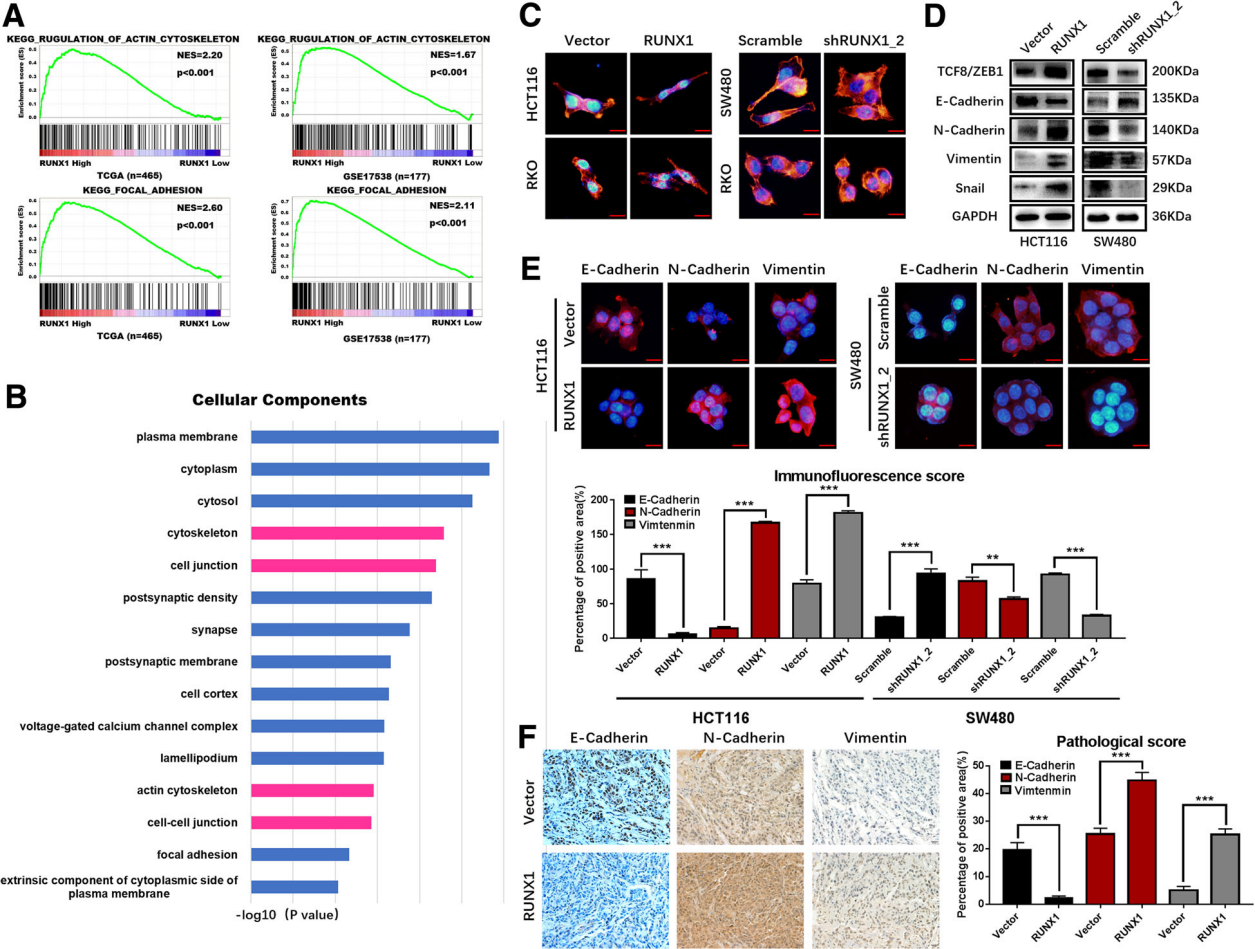
RUNX1 promotes colorectal cancer progression and metastasis via EMT. **a** Relationship of actin cytoskeleton and focal adhesion regulation with RUNX1 expression was shown using TCGA (n = 465) and GSE17538 (*n* = 177). **b** Enrichment analysis of GO-Cellular components showed cytoskeleton、cell junctions、actin cytoskeleton and cell-cell junction were enriched with RUNX1 expression. (Database source: Gene Transcription Regulation Database: http://gtrd.biouml.org/ and DAVID: http://david.ncifcrf.gov/). **c** Cytoskeleton detected by FITC-phalloidine staining in HCT116, RKO and SW480 cells with RUNX1 overexpressed and silencing. **d** Expression of EMT-related molecules detected by western blot in HCT116/vector, HCT116/RUNX1, SW480/scramble and SW480/shRUNX1 groups. **e** Immunofluorescene assay of E-cadherin, N-cadherin and vimentin in HCT116/vector, HCT116/RUNX1, SW480/scramble and SW480/shRUNX1 cells. Relevant immunofluorescence scores were shown. **f** E-cadherin、N-cadherin and Vimentin expression detected by IHC in HCT116/Vector and HCT/RUNX1 group. Pathological scores of each were obtained

GSEA analysis using the TCGA and GSE17538 datasets showed that RUNX1 expression is associated with regulation of the actin cytoskeleton and focal adhesion (Fig. 3a). We retrieved genes with more than 100 RUNX1 transcription factor binding sites from the Gene Transcription Regulation Database (GTRD) (http://gtrd.biouml.org/) and used the results to conduct a Gene Ontology (GO) enrichment analysis using DAVID Bioinformatics Resources 6.8 (https://david.ncifcrf.gov/). The GO-Cellular Components enrichment analysis results showed gene enrichment in the cellular components of the cytoskeleton, cell junction, actin cytoskeleton and cell-cell junction (Fig. 3b). Next, immunofluorescence labelling of F-actin was performed using phalloidin. Immunofluorescence analysis showed that the cell lines with upregulated RUNX1 were characterized by a spindle-shaped morphology and the rearrangement of F-actin fibres (Fig. 3c). The spindle shape change and loss of cell-cell contact are characteristics of the cell morphology changes that occur during EMT. Through mRNA expression correlation analysis on website GEPIA, we found that the expression of RUNX1 is positively correlated with multiple molecules of the EMT and is statistically significant, such as N-cadherin (CDH2), Vimentin (VIM), Snail and ZEB1 (Additional file 3: Figure S3A). Western blotting was used to analyze EMT markers induced by RUNX1 in the HCT116, SW480 and RKO cell lines. E-cadherin was downregulated in HCT116-RUNX1 cells and upregulated in SW480-shRUNX1 cells compared to its expression in control cells. The upregulation of ZEB1, N-cadherin and vimentin was also induced by RUNX1 in HCT116-RUNX1 cells and inhibited in SW480-shRUNX1 cells (Fig. 3d). Consistent with these results, the immunofluorescence assay results revealed decreased expression of E-cadherin, whereas the expression levels of N-cadherin and vimentin were decreased in HCT116-RUNX1 cells, and the opposite result was observed in SW480-shRUNX1_2 cells (Fig. 3e). Consistent with these findings, decreased E-cadherin and increased N-cadherin and vimentin expression levels were shown by immunohistochemistry in situ in the RUNX1-overexpressing cecum tumours of nude mice (Fig. 3f). Through mRNA expression correlation analysis on website GEPIA, we found that the expression of RUNX1 is positively correlated with multiple molecules of the MMP family and is statistically significant, such as MMP3, MMP7, MMP9 and MMP14 (Additional file 3: Figure S3C). Subsequently, we also used qPCR experiments to detect elevated expression of the above molecules in the HCT116 cell line overexpressing RUNX1 (Additional file 4: Figure S4A). These results strongly suggest that RUNX1 is involved in enhancing the metastatic capacity of CRC.

### RUNX1 enhances Wnt/β-catenin pathway activation in CRC

**Fig. 4:**
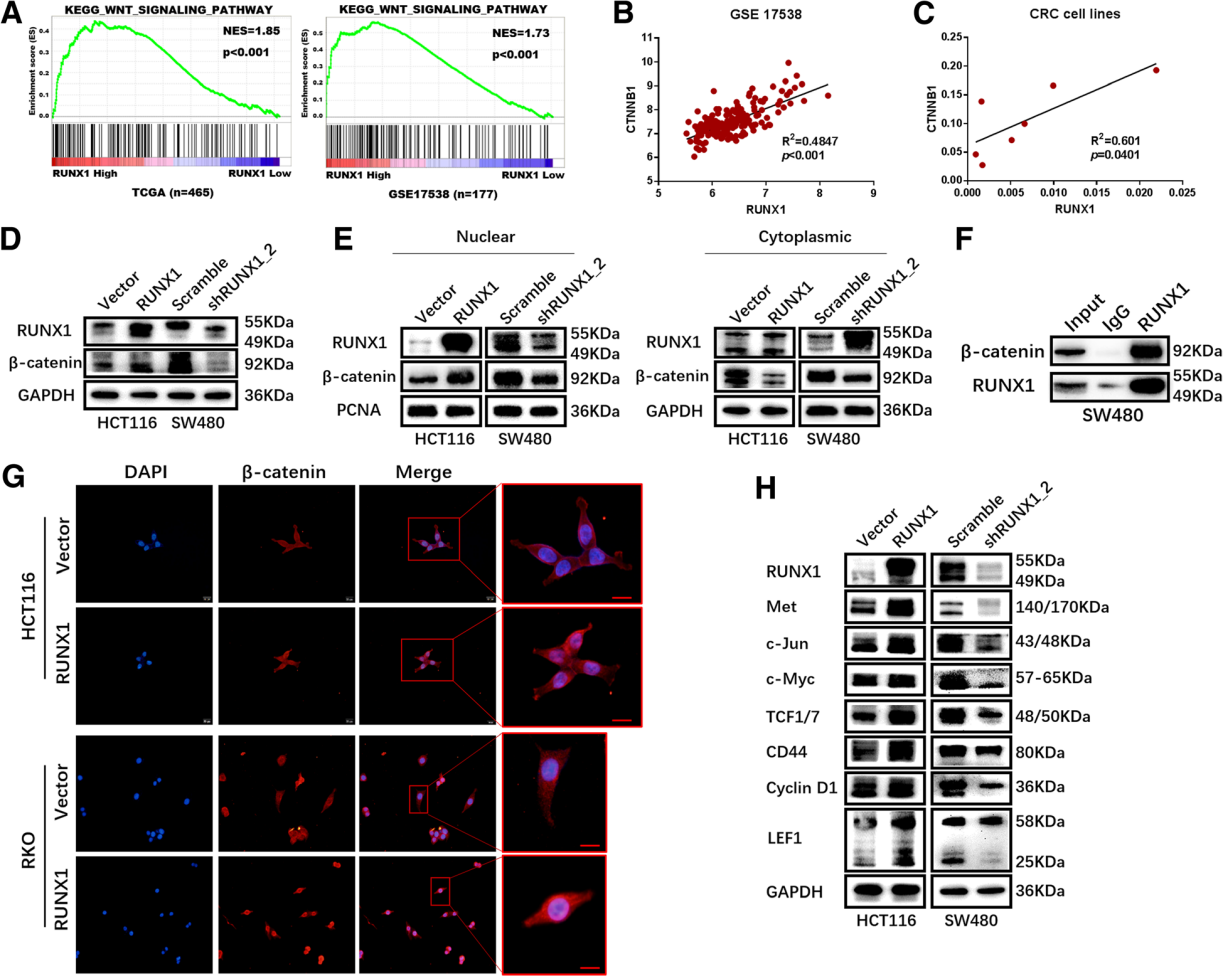
RUNX1 performing functions in CRC development via Wnt/β-catenin signaling pathway. **a** Enrichment of WNT signaling pathway with RUNX1 expression was shown using enrichment analysis of TCGA (n = 465) and GSE17538(n = 177). **b** RUNX1 expression correlates to CTNNB1 gene (β-catenin coding gene) positively, given by a colorectal cancer data set of GSE17538. **c** RUNX1 relates to CTNNB1 gene positively in different CRC cell lines. **d** Expression of RUNX1 and β-catenin protein in HCT116/Vector, HCT116/RUNX1, SW480/scramble and SW480/shRUNX1 groups. **e** Expression of nuclear or cytoplasmic RUNX1 and β-catenin protein in HCT116 and SW480 cells with RUNX1 overexpressed and silencing. The control for normalization of nuclear and cytoplasmic separation was PCNA and GAPDH, respectively. **f** Co-immunoprecipitation assay observed RUNX1 interacts with β-catenin. **g** Immunofluorescence assay of β-catenin shows higher level of nuclear β-catenin in the RUNX1-overexpressing groups. **h** Expression of RUNX1 protein and targeted genes activated by Wnt signaling pathway in HCT116/Vector, HCT116/RUNX1, SW480/scramble and SW480/shRUNX1 groups

GSEA analysis using both TCGA and the GSE17538 database showed that RUNX1 expression is associated with the Wnt signalling pathway (Fig. 4a). Next, we found a positive correlation between the expression of RUNX1 and CTNNB1, also called β-catenin, in the gene expression microarray dataset (GSE17538) (Fig. 4b) and in CRC cell lines (Fig. 4c). Through mRNA expression correlation analysis on website GEPIA, we found that the expression of RUNX1 is positively correlated with multiple molecules of the Wnt/β-catenin signalling pathway and is statistically significant, such as CTNNB1, WNT5a, CD44 and LEF1 (Additional file 3: Figure S3B). Furthermore, we verified the expression of β-catenin by Western blot and immunofluorescence analysis in lentivirus-infected cell lines, and the results showed that β-catenin expression increased after RUNX1 was overexpressed and decreased after RUNX1 was downregulated (Fig. 4d and g). Western blot analysis showed that β-catenin was upregulated in the nucleus with RUNX1 overexpression and downregulated in the nucleus with RUNX1 knockdown (Fig. 4e). A co-immunoprecipitation assay indicated that RUNX1 directly interacts with β-catenin in SW480 cells (Fig. 4f). We suggest that RUNX1 and β-catenin possibly enter the nucleus together to perform their functions. We used Western blotting to measure downstream targets, such as Met, C-Jun, C-Myc, TCF1/7, CD44, cyclin D1 and LEF1 (Fig. 4h). Our results showed that RUNX1 increased the expression of proteins, including Met, C-Jun, C-Myc, TCF1/7, CD44, cyclin D1 and LEF1, that are associated with the Wnt/β-catenin signalling pathway.

### RUNX1 targets KIT, and KIT knock down suppresses Wnt/β-catenin signalling

**Fig. 5:**
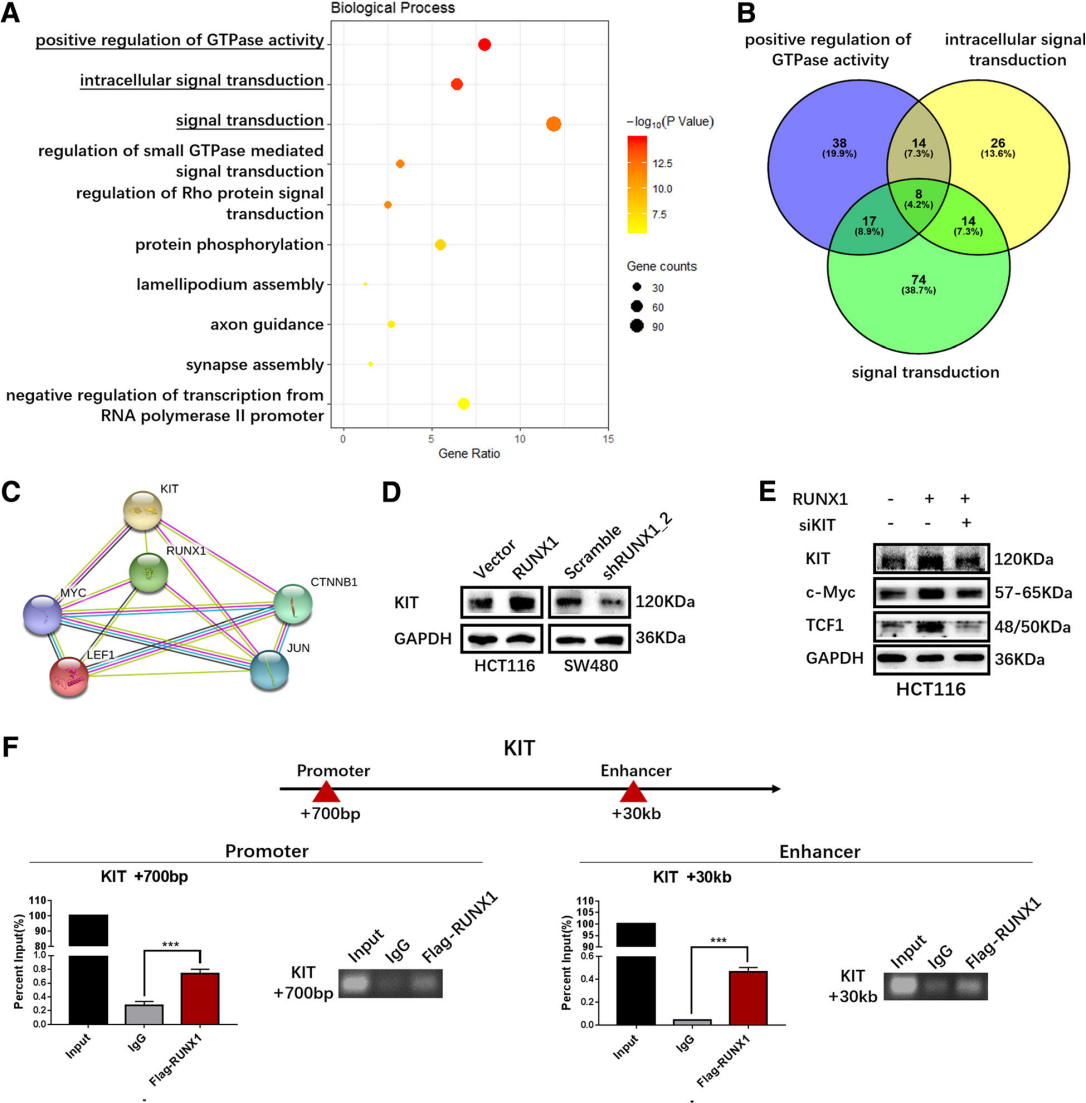
The relationship between RUNX1 and KIT. **a** Enrichment analysis of GO-Biological Precell with RUNX1 Expression was shown. **b** Interactive information of the first three biological process in Fig. 5a was shown using Venn Diagram (http://bioinfogp.cnb.csic.es/tools/venny/index.html). **c** Mutual effects between KIT and other proteins was shown using a protein association networks data set of STRING. **d** Expression of KIT protein detected by western blot in HCT116/vector, HCT116/RUNX1, SW480/scramble and SW480/shRUNX1 groups. **e** Expression of KIT protein and targeted genes activated by Wnt signaling pathway in HCT116 cells with RUNX1-overexpressed or KIT-silencing. **f** The genomic regions of KIT gene are located in two regions, one is a promoter region (+700pb) and the other is an enhancer region (+ 30 kb). Co-immunoprecipitation assay observed RUNX1 interacts with KIT both in the enhancer region and promoter region

Using genes with more than 100 RUNX1 transcription factor binding sites, a GO-Biological Process enrichment analysis was performed using DAVID Bioinformatics Resources 6.8. The three most significant biological process involved positive regulation of GTPase activity, intracellular signal transduction and signal transduction (Fig. 5a). Through a Wayne diagram using the website (http://bioinfogp.cnb.csic.es/tools/venny/index.html), we determined the intersection of these three gene sets, which contained eight molecules (Fig. 5b), including KIT, PLCB1, BCR, ARHGEF7, KALRN, MY09B, ABR and CHN2. Using the protein interaction analysis website STRING (https://version-10-5.string-db.org/cgi/input.pl), we found that KIT may be a bridge between RUNX1 and the Wnt/β-catenin signalling pathway (Fig. 5c). Moreover, Western blot results showed that the expression of KIT increased and decreased along with the expression of RUNX1 (Fig. 5d). After siRNA was used to interfere with KIT expression, the expression of several proteins, including C-Myc and TCF1, that activate the Wnt/β-catenin signalling pathway was decreased (Fig. 5e) RUNX1 transcription factor binding sites have been reported in the promoter and enhancer regions of KIT [26]. Therefore, we used ChIP-qPCR and ChIP-PCR to examine the binding sites at + 700 bp and + 30 kb from the transcription start site KIT, and the results showed that RUNX1 can bind at those two sites (Fig. 5f).

### RUNX1 is affected by the Wnt/β-catenin signalling pathway

**Fig. 6:**
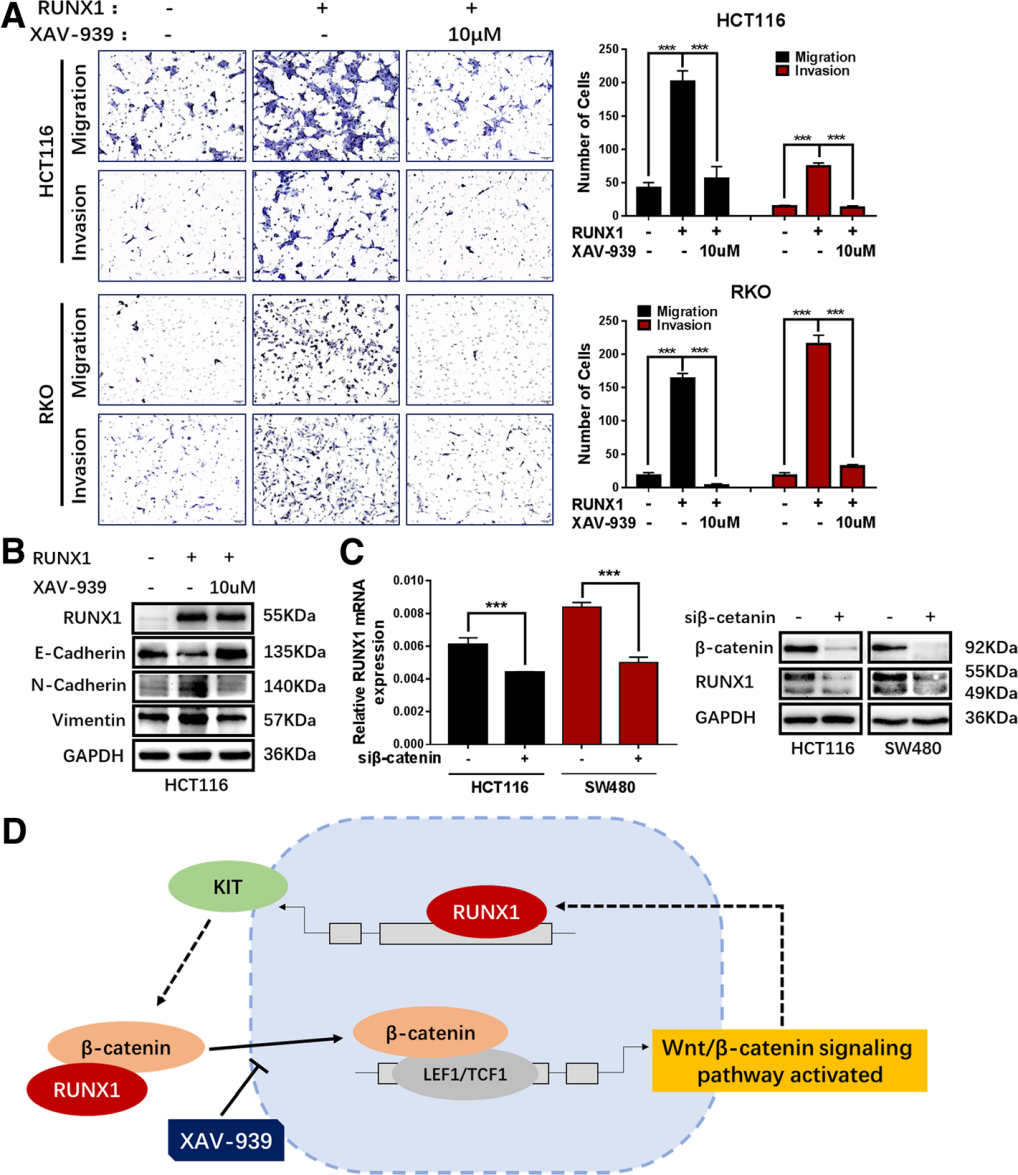
RUNX1 promotes colorectal cancer progression via RUNX1-Wnt/β-catenin signaling feedback loop. **a** Migration and invasion ability measured by Transwell assays in HCT116/RUNX1、RKO/RUNX1 、HCT116/Vector and RKO/Vector cells with the XAV939 (0/10 μM) treated. **b** Expression of EMT-related molecules by western blot in HCT116/vector and HCT116/RUNX1 cells with the XAV939 (10 μM) treated. **c** Expression of RUNX1 detected by western blot analysis and qPCR in HCT116 and SW480 cells with β-catenin silencing. **d** Hypothesized signal mechanism of RUNX1 in the development of CRC

XAV-939, a Wnt/β-catenin signalling pathway specific inhibitor, promotes β-catenin degradation. Moreover, using Transwell assays, we found that when cells were treated with XAV-939, the migration and invasion ability of HCT116 and RKO RUNX1-overexpressing cell lines was decreased (Fig. 6a). At the same time, XAV-939 can inhibit the expression changes of EMT-related molecules induced by RUNX1 (Fig. 6b). We used qRT-PCR and Western blotting to examine RUNX1 expression after treatment with siRNA of β-catenin for 48 h, and the results showed that the expression of RUNX1 decreased when the β-catenin expression was disturbed. (Fig. 6c).

## Discussion

The roles of RUNX1 have been described in many different cancers; RUNX1 is overexpressed in various epithelial tumours, especially during tumour initiation [19]. Some studies on ovarian carcinoma have suggested that RUNX1 contributes to cell proliferation, migration and invasion [27, 28]. In studies of oesophageal cancer, RUNX1a, a transcript of RUNX1, has been shown to promote cell proliferation and tumour growth [29]. Interestingly, RUNX1 functions as both an oncogene and a tumour suppressor in breast cancer [30]. RUNX1 loss promotes oestrogen receptor ER+ breast cancer epithelial cell growth and stem cell markers, but this growth promotion does not occur in ER- breast epithelial cell lines [14, 17, 18]. RUNX1 also plays dual role in gastric cancer [31–33]. Although the oncogenic role of RUNX1 in CRC has not been thoroughly examined, some studies have laterally confirmed its function [24, 34].

An unresolved question is whether RUNX1 functions to promote or suppress tumour metastasis in CRC. This report is the first direct investigation of the function and mechanism of action of RUNX1 in CRC. First, we found that RUNX1 expression was upregulated in CRC by detecting its levels in paired samples. Next, we identified RUNX1 as an oncogenic transcription factor that promotes CRC cell metastasis both in vitro and in vivo, and downregulation of RUNX1 had the opposite effect. Moreover, we found that RUNX1 promotes EMT in CRC. Mechanistically, RUNX1 promotes CRC metastasis by directly interacting with β-catenin and activating KIT transcription to promote β-catenin nucleation and the subsequent activation of the Wnt signalling pathway.

In this study, we found that RUNX1 expression was significantly increased in CRC tissues, which is consistent with findings from TCGA and the Gene Expression Omnibus (GEO) database. Furthermore, the expression of RUNX1 was positively associated with CRC metastasis. Therefore, it can be concluded that RUNX1 acts as an oncogene or risk factor in CRC tumorigenesis, which further confirms previous research [24, 25, 34]. The results of our in vitro and in vivo experiments showed that RUNX1 increased CRC the migration and invasion capacities of cell lines, and this effect is related to EMT.

It is noteworthy that some research has verified that the loss of RUNX1 expression in ER+ mammary epithelial cells increases β-catenin, deregulates mitosis and stimulates cell proliferation and expression of stem cell markers [18]. However, in complementary experiments, we also found that RUNX1 could affect the expression of AXIN1 protein, but no clear positive correlation between the two molecules was observed (Additional file 4: Figure S4B). Objectively, other results suggest a positive correlation between RUNX and the Wnt/β-catenin signalling pathway [20–23]. The RUNX1 gene can be a Wnt4 signalling target, and RUNX1 and Wnt4 are mutually interdependent in their expression in ovary cells [20]. A study involving leukaemia reported that Wnt/β-catenin signalling increases the expression of the ETO and RUNX1 genes in human haematopoietic progenitors [21], and the distal P1-RUNX1 promoter is a direct transcriptional target of Wnt/β-catenin signalling [22]. All of the abovementioned results are consistent with those of this study. Treatment with the Wnt/β-catenin pathway inhibitor XAV-939 resulted in decreases in RUNX1 expression and cell migration ability. Canonical Wnt signalling was investigated in RUNX1-deficient bone marrow stem cells, and decreases in β-catenin, LEF1, TCF1 and Wnt10b expression were found in these cells [23]. Our results also showed the corresponding conclusions that RUNX1 is positively correlated with CTNNB1 expression, and the Wnt/β-catenin pathway was activated when RUNX1 was overexpressed and inhibited when RUNX1 was downregulated.

In many cancers, Wnt/β-catenin signalling is constitutively active and promotes EMT [35]. Nuclear β-catenin binds to members of the TCF/LEF family of transcription factors to promote EMT. LEF1 is another key transcription factor that can directly induce EMT by repressing E-cadherin [36, 37]. In this study, we found that RUNX1 promotes β-catenin expression and activates Wnt signalling, and some transcription factors, such as LEF1, which can repress E-cadherin, are also activated. Previous studies have shown that MMPs are associated with the occurrence of EMT [38], while MMPs are regulated by Wnt/β-catenin signalling pathways [39–42].

As a transcription factor, RUNX1 may regulate the transcription of target genes in many ways. Thirty percent of all RUNX1 binding sites are intergenic, indicating its diverse roles in promoter and enhancer regulation and suggesting additional functions of RUNX1 [30]. We validated two strong RUNX1 binding regions by ChIP-qPCR and ChIP-PCR in the first intron of KIT: one at + 700 bp and another at + 30 kb from the transcription start site [26]. Moreover, our results showed that the expression of KIT increased with the overexpression of RUNX1 and decreased with the reduced expression of RUNX1. KIT signalling may play a growth-stimulatory role in colon cancer [43]. Furthermore, KIT activation may be associated with tumour aggressiveness via the activation of Wnt/β-catenin signalling [44]. We observed that the Wnt pathway was inhibited when KIT expression was disturbed by siRNA transfection, which may be the mechanism by which RUNX1 regulates the Wnt/β-catenin signalling pathway.

In conclusion, the current study illustrates that RUNX1 functions as an oncogene to facilitate metastasis and EMT in CRC by directly interacting with β-catenin and activating KIT transcription to enhance the Wnt/β-catenin signalling pathway (Fig. 6d). These findings enhance our understanding of CRC metastasis. In addition, RUNX1 is downstream of the Wnt pathway and is regulated by Wnt/β-catenin. After further research, RUNX1 may become a vital prognostic biomarker and an effective target for anti-metastasis therapies for CRC.

## Conclusions

In summary, our results highlight a crucial role for RUNX1 in the regulation of tumour metastasis in CRC by activating the Wnt/β-catenin signalling pathway and EMT, and RUNX1 might be regarded as a potential prognostic marker and as an effective therapeutic target for CRC.

## Additional files

Additional file 1: **Figure S1.** A. Relapse free survival with low/high RUNX1 gene expression was analyzed using a colorectal cancer data set of GSE14333/17536/31595/39582. B. Overall survival with low/high RUNX1 gene expression was analyzed using a colorectal cancer data set of GSE17536/39582. C. Expression of RUNX1 mRNA and protein detected by qPCR and western blot in colorectal cancer cell lines. (TIF 2047 kb)

Additional file 2: **Figure S2.** A. Migration and invasion ability determined by Transwell assay in HCT116, RKO and SW480 cells with RUNX1 overexpressed or silencing. B. Migration ability detected by Wound healing in HCT116, RKO and SW480 cells with RUNX1 overexpressed or silencing. C. External whole-body、colon and ileocecus images of mice by orthotopic injection of HCT116/RUNX1 and HCT116/Vector cells. HE staining of orthotopic colorectal cancer were also exhibited. (TIF 11573 kb)

Additional file 3: **Figure S3.** A. Positive correlation between RUNX1 expression and EMT targeted genes. B. Positive correlation between RUNX1 expression and CTNNB1、WNT5A、CD44 and LEF1 were observed. C. Relationship between RUNX1 expression and MMP3、MMP7、MMP9 and MMP14 were also found positively in GEPIA (GEPIA: http://gepia.cancer-pku.cn/). (TIF 3666 kb)

Additional file 4: **Figure S4.** A. The mRNA expression level of MMP3、MMP7、MMP9 and MMP14 were higher in HCT116/RUNX1 group than that in HCT116/vector group. B. The protein level of AXIN1 detected by western blot in HCT116 and SW480 cells with RUNX1 overexpression or silencing. (TIF 431 kb)

Additional file 5: **Table S1.** Primer Sequences Used for Real-time PCR (5′ to 3′). (DOC 31 kb)

Additional file 6: **Table S2.** Chip primer sequenced used for quantitative real-time PCR. (DOC 29 kb)

Additional file 7: **Table S3.** Relationship between RUNX1 mRNA expression and the clinicpathological features of CRC patients. (DOCX 14 kb)

## Data Availability

All data analysed during this study are included in this manuscript.

## Abbreviations

CHOL: Cholangiocarcinoma
COAD: Colon adenocarcinoma
ESCA: Esophageal carcinoma
LIHC: Liver hepatocellular carcinoma
PAAD: Pancreatic adenocarcinoma
READ: Rectum adenocarcinoma
STAD: Stomach adenocarcinoma
