# Supplementary figures and images for: RUNX1 promotes tumour metastasis by activating the Wnt/β-catenin signalling pathway and EMT in colorectal cancer
Source: J Exp Clin Cancer Res. 2019 Aug 1;38:334. doi: 10.1186/s13046-019-1330-9 (PMC6670220; doi:10.1186/s13046-019-1330-9)

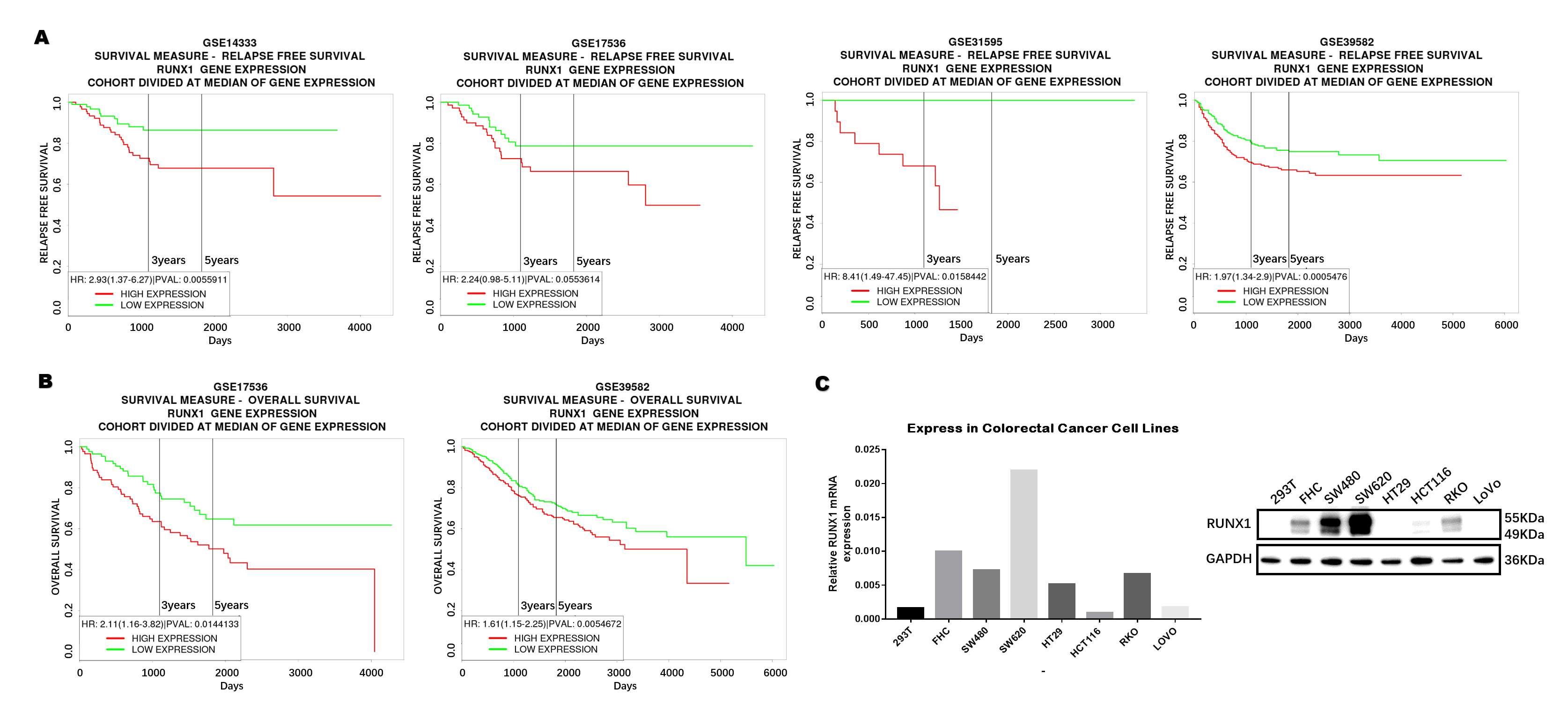

Supplement: Supplementary file 1 — Figure S1. A. Relapse free survival with low/high RUNX1 gene expression was analyzed using a colorectal cancer data set of GSE14333/17536/31595/39582. B. Overall survival with low/high RUNX1 gene expression was analyzed using a colorectal cancer data set of GSE17536/39582. C. Expression of RUNX1 mRNA and protein detected by qPCR and western blot in colorectal cancer cell lines. (TIF 2047 kb) [file 13046_2019_1330_MOESM1_ESM.tif]

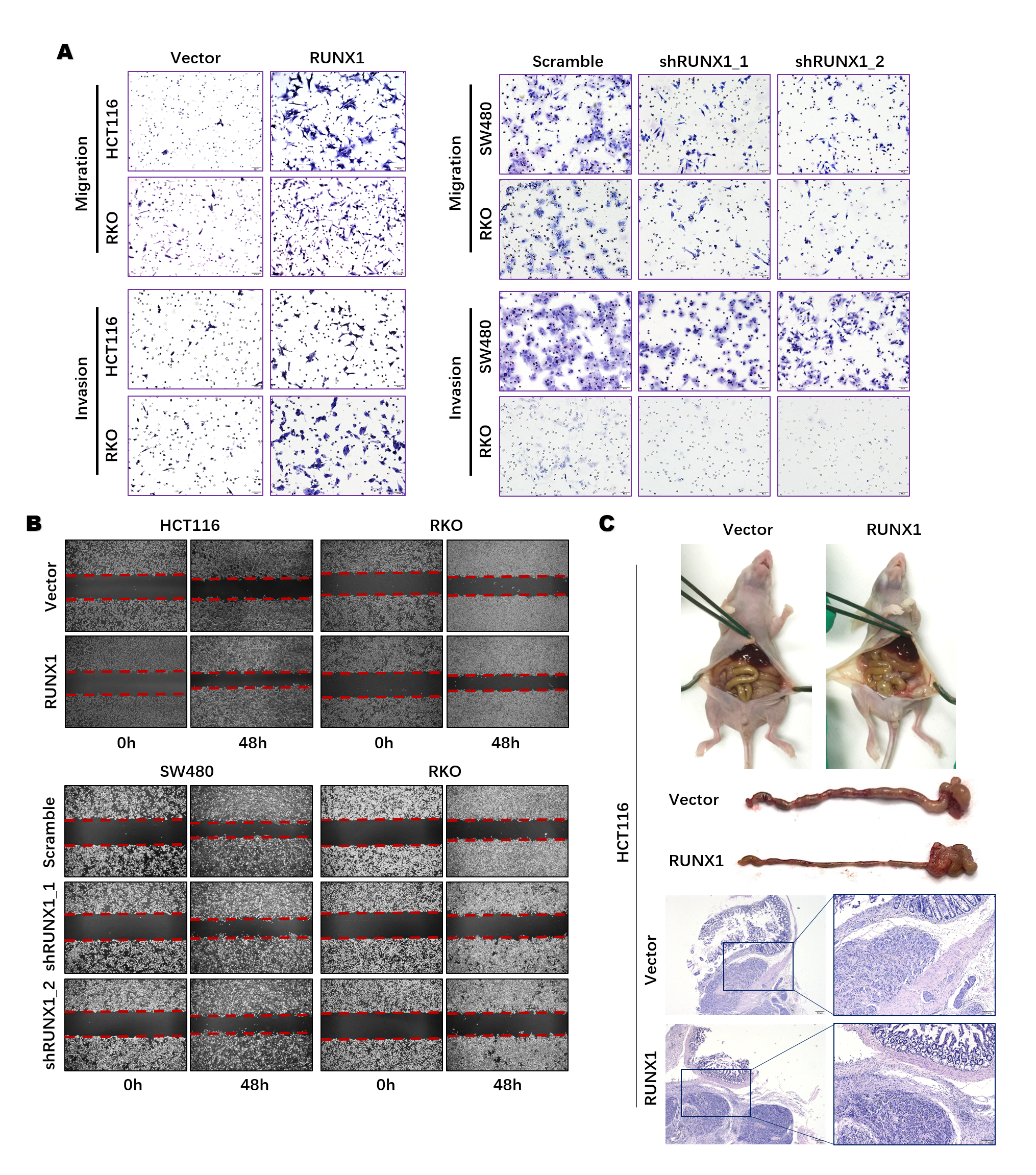

Supplement: Supplementary file 2 — Figure S2. A. Migration and invasion ability determined by Transwell assay in HCT116, RKO and SW480 cells with RUNX1 overexpressed or silencing. B. Migration ability detected by Wound healing in HCT116, RKO and SW480 cells with RUNX1 overexpressed or silencing. C. External whole-body、colon and ileocecus images of mice by orthotopic injection of HCT116/RUNX1 and HCT116/Vector cells. HE staining of orthotopic colorectal cancer were also exhibited. (TIF 11573 kb) [file 13046_2019_1330_MOESM2_ESM.tif]

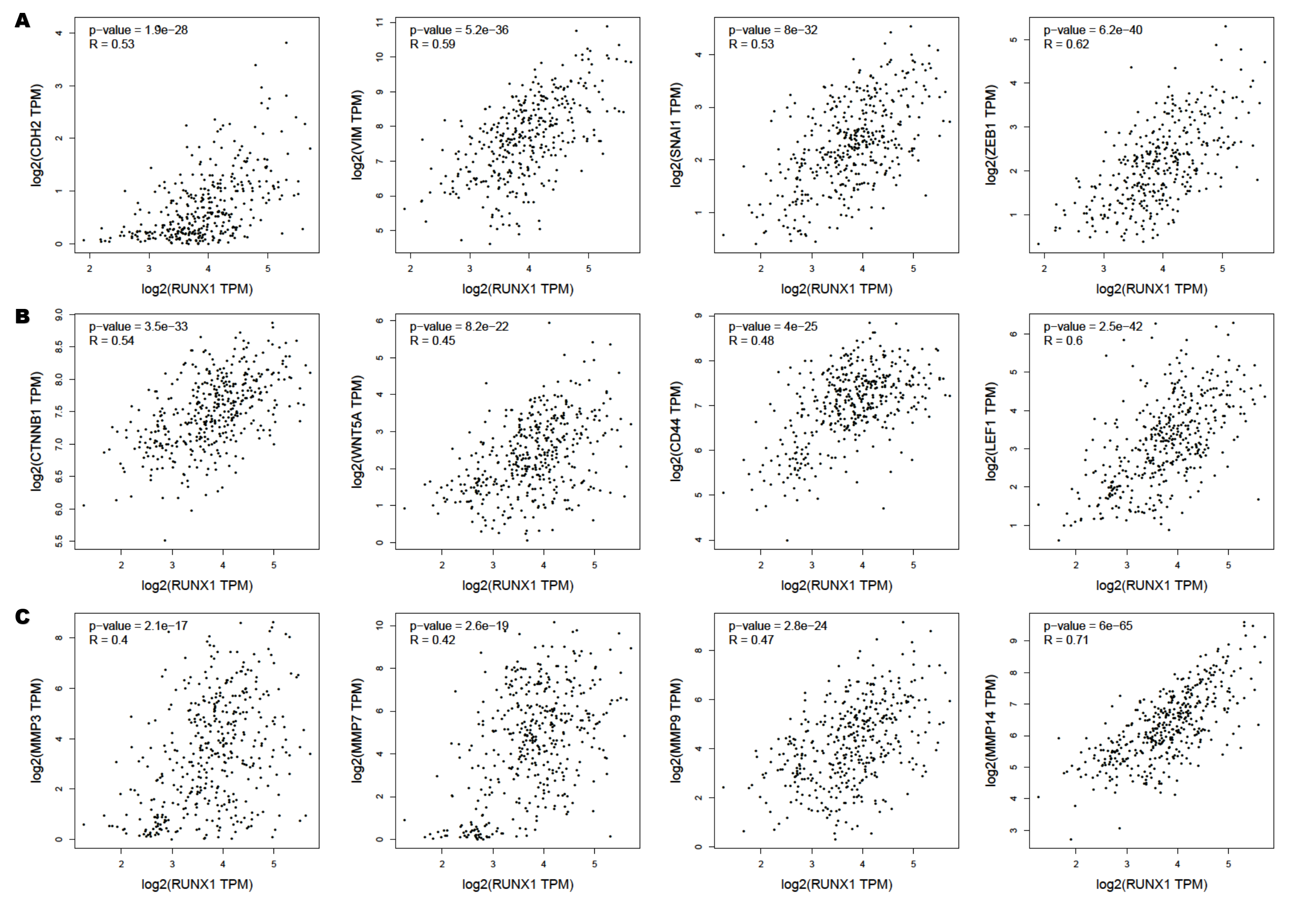

Supplement: Supplementary file 3 — Figure S3. A. Positive correlation between RUNX1 expression and EMT targeted genes. B. Positive correlation between RUNX1 expression and CTNNB1、WNT5A、CD44 and LEF1 were observed. C. Relationship between RUNX1 expression and MMP3、MMP7、MMP9 and MMP14 were also found positively in GEPIA (GEPIA: http://gepia.cancer-pku.cn/). (TIF 3666 kb) [file 13046_2019_1330_MOESM3_ESM.tif]

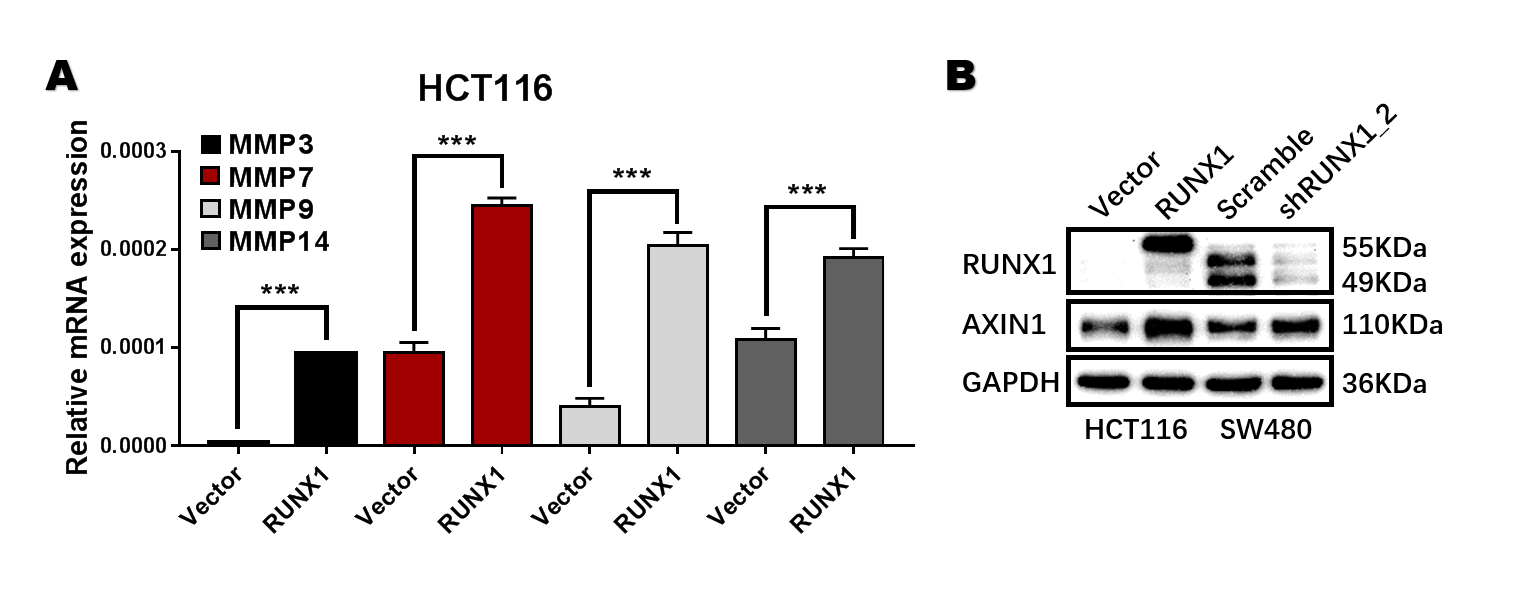

Supplement: Supplementary file 4 — Figure S4. A. The mRNA expression level of MMP3、MMP7、MMP9 and MMP14 were higher in HCT116/RUNX1 group than that in HCT116/vector group. B. The protein level of AXIN1 detected by western blot in HCT116 and SW480 cells with RUNX1 overexpression or silencing. (TIF 431 kb) [file 13046_2019_1330_MOESM4_ESM.tif]
