# Supplementary material for: RUNX1 promotes tumour metastasis by activating the Wnt/β-catenin signalling pathway and EMT in colorectal cancer
Source: J Exp Clin Cancer Res. 2019 Aug 1;38:334. doi: 10.1186/s13046-019-1330-9 (PMC6670220; doi:10.1186/s13046-019-1330-9)
Supplement: Supplementary file 5 — Table S1. Primer Sequences Used for Real-time PCR (5′ to 3′). (DOC 31 kb) [file 13046_2019_1330_MOESM5_ESM.doc]

**Table S1.** Primer Sequences Used for Real-time PCR (5' to 3')

| **Gene** | **Forward primer** | **Reverse primer** |
| --- | --- | --- |
| GAPDH | GACTCATGACCACAGTCCATGC | AGAGGCAGGGATGATGTTCTG |
| RUNX1 | CACTGTGATGGCTGGCAATGATG | CTCTGTGGTAGGTGGCGACTTG |
| CTNNB1 | AAAGCGGCTGTTAGTCACTGG | CGAGTCATTGCATACTGTCCAT |
| MMP3 | GGTGTGGAGTTCCTGATGTTGGTC | AGCCTGGAGAATGTGAGTGGAGTC |
| MMP7 | AAATGCCAACAGTTTAGAAGCC | ATTATTTCTATGACGCGGGAGT |
| MMP9 | CAGTACCGAGAGAAAGCCTATT | CAGGATGTCATAGGTCACGTAG |
| MMP14 | CAAGATTGATGCTGCTCTCTTC | ACTTTGATGTTCTTGGGGTACT |
