# Supplementary material for: RUNX1 promotes tumour metastasis by activating the Wnt/β-catenin signalling pathway and EMT in colorectal cancer
Source: J Exp Clin Cancer Res. 2019 Aug 1;38:334. doi: 10.1186/s13046-019-1330-9 (PMC6670220; doi:10.1186/s13046-019-1330-9)
Supplement: Supplementary file 6 — Table S2. Chip primer sequenced used for quantitative real-time PCR. (DOC 29 kb) [file 13046_2019_1330_MOESM6_ESM.doc]

**Table S2.** Chip primer sequenced used for quantitative real-time PCR

| **Gene** | **Forward primer (5'to 3')** | **Reverse primer (5' to 3')** |
| --- | --- | --- |
| KIT-700bp | CCTCCGGGTTAGGCTTTTG | CGAAAGCAAAAAGTCTGCAA |
| KIT-30kb | TGCATTGAGCTCCCCATAGT | TGGAAATAGCATGCAGATGG |
