# Supplementary material for: RUNX1 promotes tumour metastasis by activating the Wnt/β-catenin signalling pathway and EMT in colorectal cancer
Source: J Exp Clin Cancer Res. 2019 Aug 1;38:334. doi: 10.1186/s13046-019-1330-9 (PMC6670220; doi:10.1186/s13046-019-1330-9)
Supplement: Supplementary file 7 — Table S3. Relationship between RUNX1 mRNA expression and the clinicpathological features of CRC patients. (DOCX 14 kb) [file 13046_2019_1330_MOESM7_ESM.docx]

Table S3. Relationship between RUNX1 mRNA expression and the clinicpathological features of CRC patients

|  |  | RUNX1 mRNA expression | |  |
| --- | --- | --- | --- | --- |
| Features | n | Low(30) | High(31) | p value |
| Age, years |  |  |  |  |
| ≥60 | 38 | 16 | 22 | 0.155395 |
| ＜60 | 23 | 14 | 9 |  |
| Gender |  |  |  |  |
| Male | 36 | 19 | 17 | 0.500045 |
| Female | 25 | 11 | 14 |  |
| Intestinal section |  |  |  | 0.705191 |
| colon | 29 | 15 | 14 |  |
| rectum | 32 | 15 | 17 |  |
| AJCC stage |  |  |  | 0.025307 |
| 1-2 | 36 | 22 | 14 |  |
| 3-4 | 25 | 8 | 17 |  |
| T stage |  |  |  | 0.024098 |
| 1-2 | 13 | 10 | 3 |  |
| 3 | 42 | 18 | 24 |  |
| 4 | 6 | 2 | 4 |  |
| N stage |  |  |  | 0.228226 |
| 0 | 39 | 22 | 17 |  |
| 1 | 17 | 7 | 10 |  |
| 2 | 5 | 1 | 4 |  |
| M stage |  |  |  | 0.003308 |
| 0 | 50 | 29 | 21 |  |
| 1 | 11 | 1 | 10 |  |

According to the median expression of RUNX1 mRNA, the patients are divided into two groups: high expression group and low expression group.
